# Glances at the Hospitals

**Published:** 1900-07-07

**Authors:** 


### GLANCES AT THE HOSPITALS.

### THE EDINBURGH ROYAL INFIRMARY.

On October 1st, 1898, there were 658 patients in this infirmary, and between that date and October 1st, 1899, there were 9,036 admitted, making a total of 9,694 under treatment during the year. Of these 4,309 were discharged cured, 3,341 discharged relieved, 676 left for various other reasons, and 713 died, leaving 655 under treatment. The medical and surgical cases were nearly the same, but the surgical showed a slight increase. The numbers show an increase of 260 over those of the previous year. The average length of residence was nearly 27 days, and the highest number reached in any one day was 739; 5,233 of the patients came from Edinburgh and Leith, and 3,803 from the country. The percentage of deaths to cases treated was 7·4, but it should be mentioned that 203 patients died within 48 hours of admission, and deducting these would reduce the percentage of deaths to 5·3. The out-patients numbered 29,439, being a decrease of 510 on the previous year. The total ordinary receipts amounted to £30,739, being an increase over those for 1898 of £355. The ordinary expenditure amounted to £43,882, showing an increase of nearly £2,000. Part of this increase was owing to an improvement in the diet, and part owing to the increased price of coal, and to the nurses' home of rest. The annual cost of each bed occupied was £61 14s. The extraordinary receipts, consisting of legacies and donations, amounted to £68,000, and the extraordinary expenditure amounted to £5,614. The average number of nurses and probationers employed during the year was 190, and it is a proof of the popularity of the nursing profession that there were received during 1898 not fewer than 578 applications; while in the domestic department it was impossible to keep the staff up to its full numbers. Three new pavilions are being added to the infirmary—one for diseases of the eye, one for diseases of the ear, and one for diseases of women. There is a total and most regrettable absence of all medical details in this report. It would have been most interesting to hear the results of treatment in many diseases in this famous hospital, and to learn the number of medical students who attend its *clinique.* With these exceptions it is clearly arranged.

### THE ROYAL INFIRMARY, NEWCASTLE-UPON-TYNE.

From the one hundred and forty-ninth report we learn that 4,180 cases were admitted during 1899, and that the daily average number resident was 268. Both of these numbers show a decrease on those for 1898. The average length of residence was about 23½ days. There were 353 deaths, being at the rate of 8·44 per cent. on the number admitted, but it should be stated that 99 of the deaths occurred within 24 hours of admission, being cases of severe accident or of acute or advanced diseases beyond hope of amelioration. The out-patients reached the considerable total of 25,049, and in addition to this there were 16,320 casuals. Adding all the numbers we reach a grand total of 45,770. About 17,500 patients are treated annually in the other charitable institutions of Newcastle, and as the population of the town does not exceed 200,000 we arrive at the conclusion that one in every three of the population received gratuitous medical aid in 1899. We are aware that some of the patients come from the country districts in Northumberland and Durham, but we have taken no note of those under treatment in the Poor Law infirmaries. The total ordinary income of the infirmary amounting to £13,000, being an increase of £326 on that of the previous year. The committee say they have much pleasure in reporting that the workmen's subscriptions were £710 above those of the previous year, and amounted to £4,718. Nevertheless the expenditure in the hospital showed an excess over income of £2,635. The average cost of each in-patient was £3 14s. 10d., and the gross cost per bed occupied was £58 6s. 10d. If the cost of out-patients and the bank interest be omitted the cost per bed would be £53 16s. 7d. The medical report and the medical and surgical tables are very carefully prepared. The in-patients may be divided into 1,339 medical and 2,841 surgical cases. Among the out-patients were 2,836 ophthalmic cases, 1,156 cases of skin diseases, and 1,425 throat and ear cases. In addition to these special departments there were nearly 6,000 ordinary medical and surgical cases. Among the in-patients pneumonia and pleurisy show an increase over former years. There were 14 cases of cerebral tumour, and 24 of peripheral neuritis. One hundred and fifty cases of heart disease and 30 of aneurism were under treatment. There were 2,283 operations performed. Of these, 105 were major operations, and only six of these were followed by death. We note that there were 51 cases of ovariotomy with only one death; 109 operations for the radical cure of hernia, with only one death; and 15 knee-joints were opened without a death. We have gone somewhat more fully than usual into these statistics as there is a medical school attached to the infirmary, and we wished to show what ample opportunities for practical study the students possess. The medical staff consists of 29 physicians and surgeons. The secretary is Mr. Oliver, and the superintendent of nurses, Miss Emily Aston.

### THE ESSEX AND COLCHESTER GENERAL HOSPITAL.

During the year 1899 583 patients were admitted, and this number included 163 accidents or emergency cases. The out-patients numbered 1,973. This is the highest number reached for several years, and the total number of attendances of these patients was 5,832. There was also an increase in the cases in the casualty-room, where 573 patients were attended to. The finances of the hospital are in a satisfactory condition, as the managers are able to report that the income exceeded the expenditure by £208; but the alterations and improvements in the wards exceeded the sum subscribed for these purposes, and it was found necessary to sell out stock to meet the extra expenses. The average cost per in-patient was £3 19s. 8d.

